# Dosing Strategies for Improving the Risk-Benefit Profile of Ponatinib in Patients With Chronic Myeloid Leukemia in Chronic Phase

**DOI:** 10.3389/fonc.2021.642005

**Published:** 2021-03-16

**Authors:** Fausto Castagnetti, Fabrizio Pane, Gianantonio Rosti, Giuseppe Saglio, Massimo Breccia

**Affiliations:** ^1^ Azienda Ospedaliero-Universitaria di Bologna, Istituto di Ematologia “Seràgnoli”, Bologna, Italy; ^2^ Department of Experimental, Diagnostic and Specialty Medicine (DIMES), University of Bologna, Bologna, Italy; ^3^ Dipartimento di Medicina Clinica e Chirurgia, Università di Napoli Federico II, Napoli, Italy; ^4^ IRCCS Istituto Romagnolo per lo Studio dei Tumori (IRST) “Dino Amadori”, Meldola, Italy; ^5^ Division of Hematology & Internal Medicine, Department of Clinical & Biological Sciences of the University of Turin, ‘San Luigi Gonzaga’ University Hospital, Orbassano, Italy; ^6^ Hematology, Department of Translational and Precision Medicine, Sapienza University, Rome, Italy

**Keywords:** chronic myeloid leukemia, dosing regimens, low-dose, ponatinib, risk-benefit profile, treatment algorithm

## Abstract

The treatment of chronic myeloid leukemia (CML) has been advanced by the development of small-molecule tyrosine kinase inhibitors (TKIs), which target the fusion protein BCR-ABL1 expressed by the Philadelphia chromosome. Ponatinib is a 3rd generation TKI that binds BCR-ABL1 with high affinity and inhibits most BCR-ABL1 mutants, including the T315I mutation. The approved starting dose of ponatinib is 45 mg once daily (full dose), however, the need for a full dose, especially in patients with dose adjustments due to tolerability problems, remains undemonstrated. Lower starting doses of ponatinib (30 mg or 15 mg once daily) for patients “with lesser degrees of resistance or multiple intolerances, especially those with an increased cardiovascular risk profile” has been recommended by the 2020 European LeukemiaNet. However, the available literature and guidance on the use of ponatinib at low dosage are limited. The objective of this paper is to describe how we select ponatinib dosage for CML patients in chronic phase in our clinical practice based on the available evidence and our clinical experience. We propose dosing regimens for the optimal starting dose for six generic cases of CML patients in chronic phase eligible for the switch to ponatinib and provide an algorithm to guide ponatinib dosing during treatment.

## Introduction

The development, over the past two decades, of small-molecule tyrosine kinase inhibitors (TKIs) targeting the fusion protein BCR-ABL1 expressed by the Philadelphia chromosome (Ph) has considerably advanced the treatment of chronic myeloid leukemia (CML) and Ph+ acute lymphoblastic leukemia (ALL). Among several potential mechanisms underlying the emergence of resistance to 1^st^ or 2^nd^ generation TKIs, point mutations in the BCR-ABL1 active site is one of the most relevant and actionable: the presence of mutation can impair the activity of specific TKIs, leading to treatment failure (1).

Ponatinib, a 3^rd^ generation TKI, was designed to bind the kinase domain of unmutated and mutated BCR-ABL1 with high affinity (2). Ponatinib also inhibits the T315I mutation of BCR-ABL1, which occurs in 5%–10% of resistant patients and suppresses the action of all other approved TKIs (2, 3).

Based on the initial efficacy results of the pivotal phase 2 PACE (Ponatinib Ph+ ALL and CML Evaluation) trial (2), ponatinib was approved in December 2012 by the United States Food and Drug Administration (FDA) *via* an accelerated approval program (4). However, due to an increased incidence of arterial occlusion events reported in the ongoing PACE trial, ponatinib was temporarily removed from the market in October 2013 and recommendations for dose reduction were issued (4). Regulatory approval of ponatinib, albeit with restrictions on the patient indication, was re-established in January 2014. Patients with pre-existing risk factors were shown to have a higher risk of cardiovascular events; in particular, patients with ≥2 risk factors had the highest relative risk [2.2 (95% confidence interval, 1.5–3.3)] (5).

Ponatinib is currently indicated by the FDA for the treatment of adult patients with chronic phase, accelerated phase, or blast phase CML or with Ph+ ALL for whom no other TKI is indicated, and for all adult patients with CML or Ph+ ALL carrying the T315I mutation (4). In Europe, ponatinib is approved by the European Medicines Agency (EMA) in adult patients with chronic phase, accelerated phase, or blast phase CML or with Ph+ ALL who are resistant or intolerant to dasatinib or nilotinib and for whom imatinib is not clinically appropriate, and for all patients carrying the T315I mutation (6). The 2020 updated recommendations issued by the European LeukemiaNet (ELN) recommend ponatinib in patients with resistance to a 2^nd^ generation TKI (dasatinib, nilotinib, and bosutinib), even without specific mutations, unless its use is precluded by the presence of cardiovascular risk factors (7).

The approved starting dose of ponatinib is 45 mg once daily (full dose) (4, 6). This dose was established in a phase 1, dose-escalation study, the primary objective of which was to determine the maximum tolerated dose, defined as the dose causing a dose-limiting toxic effect in no more than one in six patients (3). This study also found that at daily doses of ≥30 mg, the trough plasma concentration was >40 nM, a concentration at which ponatinib inhibited all BCR-ABL1 mutants tested and suppressed the emergence of BCR-ABL1 mutations in preclinical studies. However, as stated in the FDA prescribing information, the optimal dose of ponatinib has not yet been identified (4), and the need for a full dose, especially in patients with dose adjustments due to tolerability problems, remains undemonstrated (8). The 2020 ELN guidelines recommend a lower starting dose, 30 mg or 15 mg once daily, for the great majority of patients in chronic phase, especially those with an increased cardiovascular risk profile, with dose increases only if needed; the 45 mg once daily starting dose is recommended only for patients with the T315I mutation, compound mutations, or patients with progression to advanced-phase disease (7).

Both the FDA and EMA labels state that dose reductions should be considered in patients with a major cytogenetic response (4, 6).

In the treatment of CML it is common practice to administer TKIs indefinitely, which can be associated with a substantial burden to patients in terms of drug-related adverse events and decreased quality of life; elevated treatment costs are also an issue. Recently available evidence suggests that TKI dose-reduction strategies, in patients with a stable major molecular response (MMR) who are regularly monitored for BCR-ABL1, are safe and may constitute a valuable option for optimizing TKI-based therapy (9, 10). However, studies evaluating dose-reduction strategies with ponatinib are limited and practical indications to guide clinicians in the selection of the starting dose of ponatinib are lacking.

The objective of this paper is to describe how we select ponatinib dosage for CML patients in chronic phase in our clinical practice, based on the available evidence and our experience. To this purpose, we will first review the available data on ponatinib dose reduction then discuss the optimal starting dose for six generic cases of CML patients in chronic phase eligible for the switch to ponatinib. An algorithm for the treatment of these cases with ponatinib will also be provided.

## Reported Outcomes With Low-Dose Ponatinib

The available literature on the use of ponatinib at a low dosage is limited and there are only a few clinical publications available, mainly concerning real-life experiences. The optimal risk-benefit profile of ponatinib at doses lower than the licensed starting dose of 45 mg/day was evaluated in the PACE trial (5, 11), and is under investigation in the ongoing open-label phase 2 OPTIC (Optimizing Ponatinib Treatment In CML) trial (NCT02467270) (12, 13).

In the PACE trial, ponatinib dose reductions from the starting dose of 45 mg/day to 30 mg/day or 15 mg/day were implemented per protocol to manage adverse events or proactively in October 2013 to decrease the risk of arterial occlusive events (5). For chronic-phase CML patients with a major cytogenetic response, a decrease to 15 mg/day was recommended; for chronic-phase CML patients without a major cytogenetic response, and for patients with accelerated- and blast-phase CML, a decrease to 30 mg/day was recommended. A *post hoc* analysis found that major cytogenetic responses or MMRs were sustained for 40 months in ≥90% of 270 patients with chronic-phase CML, suggesting that, irrespective of dose reductions, ponatinib offers durable and clinically meaningful outcomes in heavily pretreated chronic-phase CML patients (5).

A pooled multivariate analysis of three clinical trials (phase 1 dose-escalating study (3) phase 2 PACE trial (2), phase 3 EPIC trial (14); 671 patients in total), which investigated the impact of ponatinib dose intensity on adverse events of interest including arterial occlusive events, found that the risk of an arterial occlusive event decreased by approximately 33% for each daily 15 mg decrease of ponatinib dose (11). This analysis identified significant associations between higher ponatinib doses and higher rates of most of the adverse events considered, suggesting the existence of a causal relationship between ponatinib dose intensity and toxicity, and supporting the need for prospective studies to determine optimal ponatinib dosing regimens.

The interim analysis (IA) of the OPTIC trial reinforced the concept that an initial treatment with 45 mg daily may induce higher response rates, especially in resistant patients, but suggest that higher dose may increase the incidence of cardiovascular events, even with a short treatment duration (12, 13). OPTIC is an ongoing, multicenter, randomized phase 2 trial which is investigating three starting doses of ponatinib (45 mg, 30 mg, 15 mg) in 282 patients, 94 patients in each treatment cohort, with chronic-phase CML resistant or intolerant to at least two TKIs, or carrying the T315I mutation. Ponatinib dose was reduced to 15 mg/day for patients who initially received a 45 mg or 30 mg daily dose and achieved a ≤1% BCR-ABL1 response. At the OPTIC IA cut-off of 21 months, the median duration of exposure varied from 12 to 14 months in the three arms of the study. For patients with 12 months of follow-up, 38.7%, 27.4%, and 26.5% of patients receiving 45, 30, and 15 mg/day starting dose achieved a ≤1% BCR-ABL1 response (primary endpoint). Responses achieved at starting dose of 45 or 30 mg/day were maintained despite the dose reduction to 15 mg/day. The dose-dependency of efficacy and safety suggested an optimal benefit-risk profile may be achieved when treatment is initiated at 45 mg/day and de-escalated to 15 mg/day when BCR-ABL1 is ≤1%. The rate of adjudicated arterial occlusive events at 21 months showed a decreasing dose-dependent trend from 5.3%, 4.3%, and 1.1%, respectively, in the 45 mg/day, 30 mg/day, and 15 mg/day starting dose cohorts.

An indirect comparison between the PACE trial (n=257) and the 45 mg starting dose of the OPTIC IA (n=93), recently presented at the 2020 ASH Meeting, confirmed that a prompt dose reduction to 15 mg after achievement of MR2 does not jeopardize the efficacy of ponatinib and remarkably reduced the incidence of treatment-emergence arterial occlusive events (15). Both PACE and the OPTIC IA showed an increase over time in the ≤1% BCR-ABL1 response rate (from 42.0% to 47.1%, respectively, at 12 and 60 months for PACE, and from 47.3% to 51.6%, respectively, at 12 and 24 months in the OPTIC IA). Serious treatment-emergent adverse events (31.2% vs. 63.4%) and adjudicated arterial occlusive events (5.4% vs. 20.2%) were lower in the OPTIC IA than in the PACE trial.

The efficacy and safety profile of low-dose ponatinib in CML patients in chronic phase emerging from real-life observations appears to confirm the results of clinical trials (**Table 1**) (16–24). A lower ponatinib starting dose appears to be a beneficial strategy in selected CML patients, such as those intolerant or with a low level of resistance to other TKIs, with reported lower incidences of adverse events, no unexpected adverse events, and efficacy data in line with those from clinical trials. Notably, a retrospective analysis of 78 CML patients treated with ponatinib in the US between 2011 and 2017 revealed a decrease of cardiovascular events beginning in 2014 when the study institution implemented lower starting ponatinib doses, early dose reductions, and referral of patients to cardio-oncologists (22). Hence, dose optimization appears to be a reality that can be achieved in clinical practice.

**Table 1:** Low-dose ponatinib efficacy and safety outcomes from real-life observations.

Study	Patient population	Ponatinib starting dose per day	Median duration of treatment	Efficacy and safety outcomes
Mauro et al. (16)	CML pts	Low-dose ≤30 mg (15 pts) Standard dose >30 mg (20 pts)	11 mo (≤30 mg/d) 12 mo (>30 mg/d)	• Efficacy response similar with low- and standard-dose ponatinib (40% low-dose pts vs. 25% standard-dose pts achieved MMR or better; p = 0.344) • No significant between-group differences in AESI, including AOEs
Binotto et al. (17)	CP-CML pts resistant or intolerant to previous TKIs	45 mg (24 pts resistant to prior TKIs) 30 mg (13 pts resistant to prior TKIs) 15 mg (25 pts; 12 pts were intolerant or intolerant/resistant to prior TKIs)	21.2 mo at any dose 15 mo at 15 mg starting dose	• 55% of pts treated with 45 mg or 30 mg ponatinib achieved MMR after a median of 4.3 mo • Dose reduction to 15 mg occurred after a median of 10 mo due to AEs (73%) or to prevent toxicity and/or following response attainment (27%) • Efficacy of de-escalated ponatinib dose confirmed in CP-CML pts resistant to prior TKIs • 15 mg as starting dose conferred potential benefit in pts intolerant or with low level resistance (all intolerant and 80% of intolerant/resistant pts achieved MMR or higher) • Hematologic and CV AEs were more frequent in pts receiving ≥30 mg versus 15 mg ponatinib
Breccia et al. (18)	CP-CML pts	45 mg (17 pts) 30 mg (11 pts) 15 mg (1 pts)	12 mo	• Dose was reduced in 2 pts due to intolerance (from 45 mg to 30 mg) and in 8 pts to reduce CV risk after obtaining a cytogenetic response (from 45 mg to 30 mg and from 30 mg to 15 mg in 4 pts each) • Improved level of response vs baseline in 85.7% of pts at a median of 12 mo (11 pts achieved MMR) • No CV AEs were observed • Ponatinib appears to be efficacious and safe in CML pts who failed front-line treatment with a 2^nd^-generation TKI
Heiblig et al. (19)	CP-CML (48 pts)	Not stated	19 mo	• OS probability at 12 and 36 mo, respectively, was 95.7% (range 90.04%–100%) and 81.5% (range 70.5%–94%) • The cumulative incidence of MMR was 66.7% and 81.8%, respectively, at 6 and 18 mo • CP-CML pts reported 37 non-severe CV events and 9 severe CV events including thrombotic and non-thrombotic events
Iurlo et al. (20)	CP-CML (53 pts)	45 mg (22 pts) 30 mg (21 pts) 15 mg (9 pts) other dose (1 pt)	23.9 mo	• Interim analysis of the OITI trial • 88.6% of CP-CML pts achieved a CCyR after 6 mo ponatinib treatment and 37.5% achieved MMR • OS rates were 96.2% and 93.1%, respectively, at 12 and 24 mo • 53.6% (30 pts) had treatment-related AEs; hypertension (*n* = 6) was the most frequent
Iurlo et al. (21)	CML pts intolerant to previous TKIs	45 mg (9 pts) 30 mg (18 pts) 15 mg (25 pts)	19.2 mo	• Depth of MR increased in 21 (40.4%) pts including 6 pts with MMR and 15 pts with DMR • AEs were reported in 24 (46.2%) pts; 4 pts experienced CV AEs including one case each of AMI and ischemic stroke both occurring in pts with pre-existing CV risk factors who were initially treated with 30 mg ponatinib
Chan et al. (22)	CP-CML (51 pts)	39.65 mg (mean starting dose) Dose reduction occurred in 64.7% of CP-CML pts	14.6 mo	• 58.7% of CP-CML pts (27/46) achieved MMR • After 2014, the proportion of pts initiating ponatinib at 45 mg/d decreased substantially (29.3% vs. 86.5% before 2014) • CV events decreased from 2014 onwards following implementation of lower starting doses, early dose reductions, and referral of pts to cardio-oncologists
Iurlo et al. (23)	CP-CML pts intolerant to dasatinib	15 mg (7 pts)	9.9 mo	• Depth of MR achieved with dasatinib was maintained or increased with low-dose ponatinib; MR increased to MR4 in 2 pts • There were no serious AEs or CV thrombotic issues requiring ponatinib discontinuation; 3 pts developed hypertension
Santoro et al. (24)	CML pts with CV risk factors	Median dosage of 30 mg (45–30 mg starting dose reduced to 15 mg)	34.6 mo	• Stable or improved MR achieved with ponatinib treatment; 4 pts achieved MMR; 1 pt achieved MR4 • Despite a reduction in ponatinib dose to 15 mg, all 5 pts maintained a target MR of at least MMR • No CV AEs were reported

AEs, adverse events; AESI, adverse events of special interest; AMI, acute myocardial infarction; AOEs, arterial occlusive events; AP, accelerated phase; BP, blast phase; CCyR, complete cytogenetic response; CML, chronic myeloid leukemia; CP, chronic phase; CV, cardiovascular; d, day; DMR, deep molecular response; MR, molecular response; MMR, major molecular response; mo, months; OITI, Observational study of Iclusig^®^ (ponatinib) Treatment in patients with CML in Italy; OS, overall survival; pts, patients; yr, year.

Collectively, the available data indicate that the treatment of CML with ponatinib can be optimized by dose modification. The evidence suggests that lowering the dose of ponatinib up to 15 mg/day is a feasible strategy for improving treatment safety and tolerability while maintaining response. Based on this evidence, and our experience in the treatment of CML, in the following practice-oriented section we propose dosing regimens for initiating ponatinib in eligible patients and provide an algorithm for guiding ponatinib dosing during treatment.

## Suggested Strategies for Initiating Treatment With Ponatinib in CML Patients in Chronic Phase

**Figure 1:**
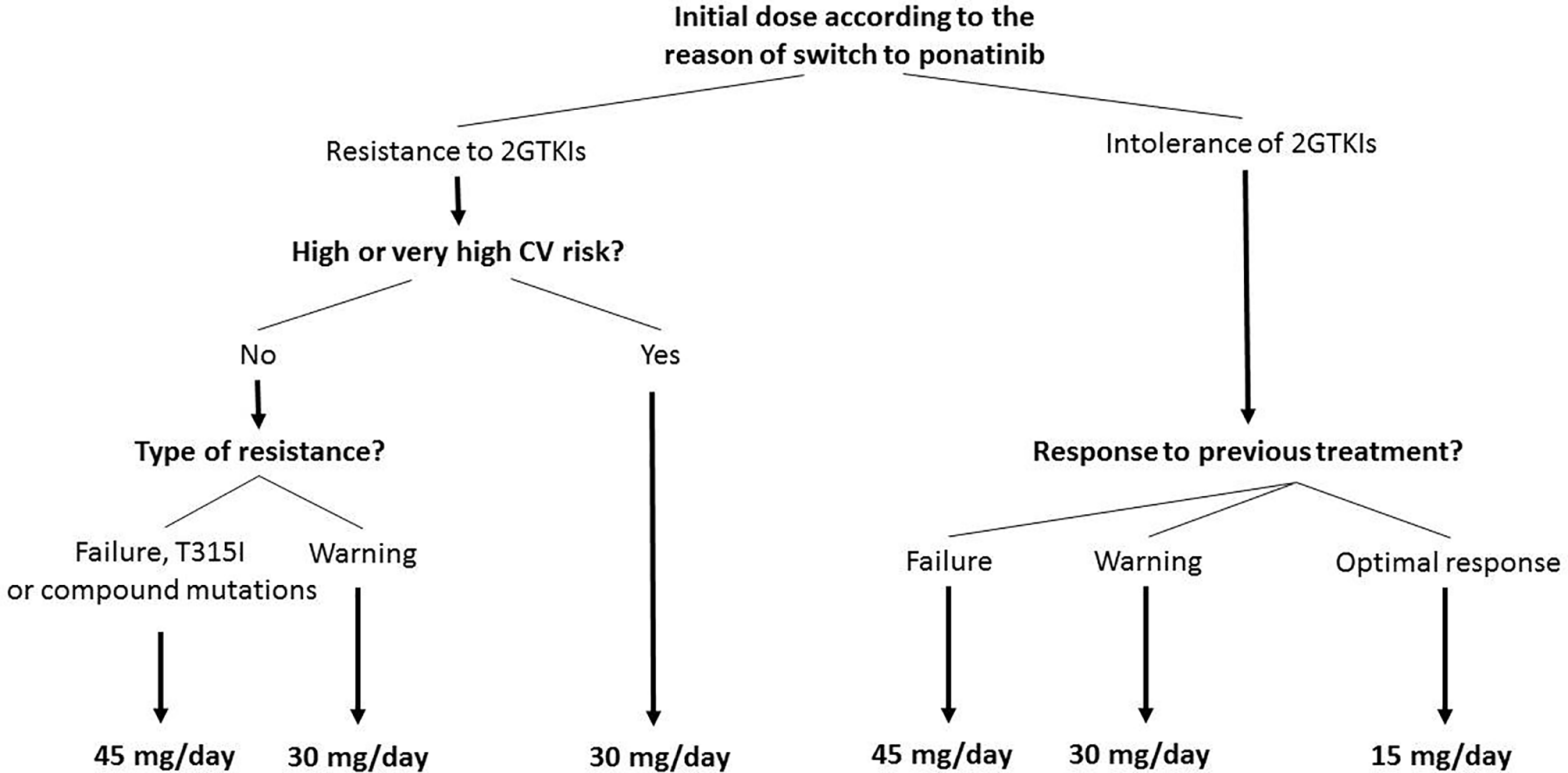
Treatment algorithm for starting doses of ponatinib in eligible patients with chronic myeloid leukemia in chronic phase. In case of hematologic toxicity of 2^nd^ generation tyrosine kinase inhibitors (2GTKIs), consider 15 mg/day as initial dose. Patients with a starting dose of 45 mg/day or 30 mg/day, should reduce to 15 mg/day upon achievement of a MR3 (MR2 in case of high or very high cardiovascular [CV] risk). Failure criteria defined according to the European LeukemiaNet (ELN) 2020 recommendations (BCR-ABL1 >10% within 1–3 months, >10% at 6 months, >1% at 12 months, and >1%, resistance mutations, high-risk additional chromosome abnormalities any time after 12 months) (7). Warning response defined according to the ELN 2020 recommendations (BCR-ABL1 >10% at 3 months, >1%–10% at 6 months, > 0.1%–1% at 12 months onwards) (7). Optimal response defined according to the ELN 2020 recommendations (BCR-ABL1 ≤10% at 3 months, ≤1% at 6 months, and ≤0.1% at 12 months onwards) (7). CV risk defined according to the 2016 European Guidelines on CV disease prevention (25).

In agreement with the approved indication of ponatinib and to current ELN recommendations (4, 6, 7), we generally consider CML patients in chronic phase eligible for ponatinib when they are resistant and/or intolerant to a 2^nd^ generation TKI; in particular, we consider a patient resistant to 2^nd^ generation TKIs if they can be defined at least as “warning” according to the 2020 ELN recommendations (BCR-ABL1 transcript >10% at 3 months, >1% at 6 months, >0.1% at 12 months or later), or when they carry the T315I mutation. Along with the response to previous TKI therapy, patient characteristics (age, comorbidities, and cardiovascular risk) should also be taken into account when selecting the starting ponatinib dose. **Figure 1** shows the algorithm we propose for the treatment of CML patients in chronic phase eligible for ponatinib and summarizes the starting dose of ponatinib that we use in six generic CML patient cases that can be encountered in clinical practice. The terms “optimal response”, “failure”, and “warning response” are defined according to the ELN 2020 recommendations (7).

Starting dose of ponatinib can be 45 mg/day (full dose), 30 mg/day, or 15 mg/day (**Figure 1**). In the PACE trial, a dose reduction has been performed in patients achieving a MCyR, while in the OPTIC trial the dose was reduced after achievement of MR2. According to most international recommendations MR3 should be considered as the optimal response, protecting patients from disease progression. Consequently, differently from the PACE and OPTIC studies, in patients initiating treatment with 45 mg/day or 30 mg/day, we suggest a dose reduction to 15 mg/day upon achievement of an MR3 response or <0.1% BCR-ABL1. It is important to underline that in the PACE trial the median times to CCyR (approximately corresponding to MR2) and MR3 were 2.9 months and 5.5 months: both PACE and the OPTIC IA highlighted the short time to molecular response on ponatinib, hence dose reduction can be very early. Considering the dose-dependent risk of cardiovascular events on ponatinib, an earlier dose reduction, namely after the achievement of MR2 (BCR-ABL1 transcript 0.1%–1%), should be considered in patients with high or very high cardiovascular risk.

Patients who fail a 2^nd^ generation TKI should start ponatinib at full dose, while patients with a “warning response” to a 2^nd^ generation TKI should start ponatinib at 30 mg/day. In patients carrying the T315I mutation or a compound mutation, ponatinib should be started at the full dose (45 mg/day). Patients who are intolerant to a 2^nd^ generation TKI without optimal response to it, should initiate ponatinib at the same dose as resistant patients, while those with an optimal response can start with ponatinib at 15 mg/day.

Finally, a reduced starting dose of 30 mg/day should be used in all patients with high or very high cardiovascular risk, regardless of the reason for the switch to ponatinib and, therefore, also following the failure of a 2^nd^ generation TKI. Cardiovascular risk must be stratified in accordance with the European Society of Cardiology Guidelines (**Table 2**) (25) and numerous consensus for the management of cardiovascular risk during treatment have been published (26, 27).

**Table 2:** Cardiovascular risk categories stratified according to the European Society of Cardiology Guidelines (25).

Risk category	Definition
Very high-risk	Patients with any of the following: • Documented clinical or unequivocal CVD on imaging o Documented clinical CVD includes previous AMI, ACS, coronary revascularization, and other arterial revascularization procedures, stroke and TIA, aortic aneurysm, and PAD o Unequivocally documented CVD includes plaque on coronary angiography or carotid ultrasound. Increase in continuous imaging parameters such as intima–media thickness of the carotid artery are NOT included • DM with target organ damage (i.e., proteinuria) or with a major risk factor (i.e., smoking, marked hypercholesterolemia, or marked hypertension) • Severe CKD (GFR <30 ml/min/1.73 m^2^) • SCORE ≥10%
High-risk	Patients with: • Markedly elevated single risk factors (i.e., cholesterol >8 mmol/L [>310 mg/dl]) or BP ≥180/110 mmHg • Most other patients with DM except for young people with type 1 DM and without major risk factors that may be at low or moderate risk • Moderate CKD (GFR 30–59 ml/min/1.73 m^2^) • SCORE ≥5% and <10%
Moderate-risk	SCORE ≥1% and <5% at 10 years
Low-risk	SCORE <1%

ACS, acute coronary syndrome; AMI, acute myocardial infarction; BP, blood pressure; CKD, chronic kidney disease; DM, diabetes mellitus; GFR, glomerular filtration rate; PAD, peripheral artery disease; SCORE, systematic coronary risk estimation; TIA, transient ischemic attack.

The proportion of patients who discontinue first- or second-line treatment with 2^nd^ generation TKIs is variable, ranging from approximately 40% to 75% depending mainly on patient characteristics, line of treatment, and the length of follow-up of published studies; around 10% to 40% of patients who switch from 2^nd^ generation TKIs do so due to an unsatisfactory response (28–34). There is no literature data to determine the number of resistant patients who have a warning response and how many have a failure. According to our algorithm, if we consider more recently published studies (where early switches are more frequent), we estimate that only a minority of patients will require 45 mg ponatinib as initial dose because of failure or T315I mutation, followed by prompt dose reduction in case of achievement of MMR.

The proposed dosing strategies and algorithm shown here, except for the starting dose of 45 mg/day, are off-label and are based entirely on the available literature and our clinical experience. Until further randomized, prospective data from clinical trials become available, a risk-benefit analysis should be applied on a case-by-case basis when treating CML patients. Indeed, anecdotal evidence suggests that clinicians start ponatinib at 45 mg/day in only a minority of cases, with most patients beginning therapy at 30 mg/day, hence dose optimization is already a reality in real-life clinical practice.

Recent recommendations from a German expert consensus panel on ponatinib proposed the following criteria supporting a ponatinib starting dose of 30 mg/day in CML patients: chronic phase, good response status, no mutations, resistance to only one TKI, intolerance despite good response, and increased cardiovascular risk (35). The strategy to start ponatinib at doses lower than 45 mg/day should be guided by a thorough evaluation of risk factors, as well as the depth and stability of molecular response, and total exposure to ponatinib; continued monitoring of response is highly recommended (26).

## Conclusions

Evidence shows that low-dose ponatinib is effective and that selected patients with CML may not need the full starting dose currently approved. The starting ponatinib dose should be indeed tailored to patient characteristics considering the response to previous TKI therapy. We propose here practical strategies for more flexible dosing of ponatinib from therapy initiation to maintenance, with the ultimate goal of improving the risk-benefit balance and the use of this potent TKI.

## Data Availability Statement

The original contributions presented in the study are included in the article/supplementary material. Further inquiries can be directed to the corresponding author.

## Author Contributions

All authors contributed to the study conception and design in equal measure. All authors contributed to the article and approved the submitted version.

## Funding

The authors declare that this study received funding from Incyte for medical writing assistance. The funder was not involved in the study design, collection, analysis, interpretation of data, the writing of this article or the decision to submit it for publication.

## Conflict of Interest

FC consultancy and honoraria: Novartis, Incyte, Pfizer, and BMS. FP received honoraria from Incyte, Novartis, BMS, and Pfizer. GR consultancy and honoraria: Novartis, BMS, Incyte, and Pfizer. GS consultancy and honoraria: Novartis, BMS, Incyte, and Pfizer. MB received honoraria from Novartis, Pfizer, Incyte, BMS/Celgene, and AbbVie.

The reviewer JHL declared a past co-authorship with the authors to the handling editor.
